# Transition-metal/Lewis acid free synthesis of acyl benzothiophenes via C-C bond forming reaction

**DOI:** 10.1186/1860-5397-3-35

**Published:** 2007-10-25

**Authors:** Sarbani Pal, Mohammad Ashrafuddin Khan, P Bindu, P K Dubey

**Affiliations:** 1 Department of Chemistry, MNR Post Graduate College, Kukatpally, Hyderabad-500072, India; 2 Department of Chemistry, JNT University, Kukatpally, Hyderabad-500072, India

## Abstract

A simple and single-step synthesis of 2- and 3-acyl substituted benzothiophenes has been described *via* environmentally benign acylation of benzothiophene with *in situ* generated acyl trifluoroacetates. Both aliphatic and aromatic carboxylic acids participated in trifluoroacetic anhydride/phosphoric acid mediated C-C bond forming reactions under solvent-free conditions affording acyl benzothiophenes in good overall yields.

## Background

**Figure 1:**
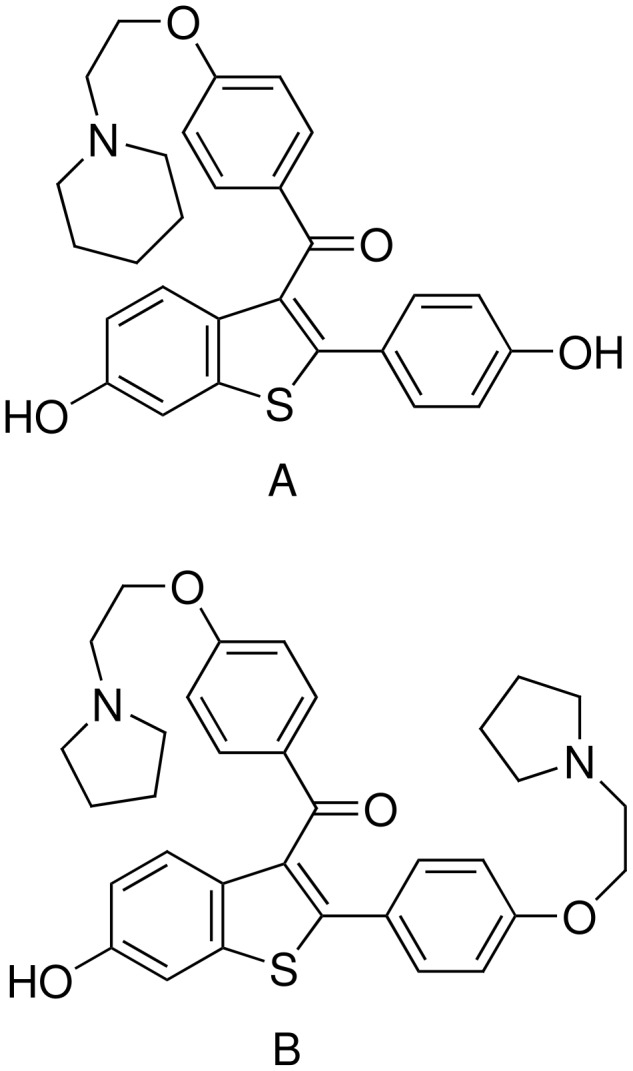
Acyl benzothiophenes of pharmacological interest.

Benzothiophene derivatives possessing an acyl group as one of the substituents on the five-membered ring are of immense medicinal value because of their promising pharmacological properties. For example, Raloxifene, [2-(4-hydroxyphenyl)-6-hydroxybenzo[*b*]thien-3-yl] [4-[2-(1-piperidinyl)ethoxy]phenyl]-methanone hydrochloride (**A**, Figure 1), is representative of a class of compounds known as selective estrogen receptor modulators[1] (SERMs) that possess estrogen agonist-like actions on bone tissues and serum lipids while displaying potent estrogen antagonist properties in the breast and uterus. A most common example of SERM is tamoxifen which has been the therapy of choice in the endocrine treatment of all stages of hormone-dependent breast cancer and in the primary and secondary chemoprevention of breast cancer. Recently, raloxifene showed promising results in clinical trial and is expected to provide an alternative to current hormone replacement therapy (HRT) that has been causally linked to breast cancer.[2] Another compound **B** (Figure 1) was identified as a thrombin inhibitor that could be utilized in the chronic treatment of thrombotic disorders.[3] Apart from medicinal interest, acyl derivatives of benzothiophene have synthetic value and a variety of diversity based benzothiophene derivatives have been prepared using these compounds. [4–12]

**Scheme 1:**
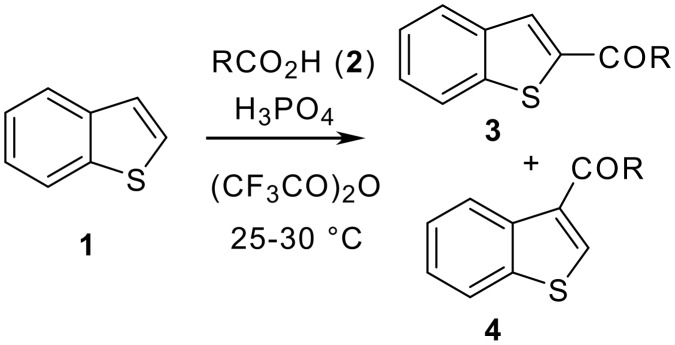
Synthesis of acyl benzothiophenes

While a number of methods are known for the synthesis of acyl benzothiophenes [13–20] many of them however, are only suitable for the preparation of certain specific compounds, namely, acetyl, propanoyl and benzoyl derivatives. Some of these syntheses require complicated reaction conditions e.g. the use of an expensive transition metal catalyst along with toxic carbon monoxide gas[13] or unstable diazo compounds,[18] or pyrophoric BuLi,[19–20] or a multistep synthesis of starting material.[14] The simplest and straightforward method for the synthesis of acyl benzothiophenes appeared to be the Friedel Crafts acylation reaction [21–24] which however, involved the use of excess AlCl_3_ and led to the formation of environmentally harmful gaseous HCl. Moreover, this protocol involved i) the use of moisture-sensitive acyl chloride, ii) the use of a large volume of chlorinated solvent and iii) the generation of aluminium waste that needs to be disposed of. Additionally, formation of unidentified side products is very common in this reaction. Therefore, the use of this method especially in large scale preparation might present major drawbacks. To overcome these difficulties we, therefore, focused on the alternative methods available in the literature [25–30] that could be utilized for the straightforward preparation of acylated benzothiophene derivatives via C-C bond forming reactions as a key synthetic step. The use of mixed anhydrides of trifluoroacetic acid for aromatic acylation has been reported as a useful alternative to the Friedel-Crafts acylation process and a variety of aromatic and aliphatic carboxylic acids have been employed successfully. Encouraged by these results, we decided to adopt a similar strategy for the acylation of benzothiophene. We anticipated that a Lewis acid/transition metal free synthesis of acyl benzothiophenes could provide an easy access to a library of benzothiophene based analogues of potential biological interest. Notably, while the use of various arenes/heteroarenes has been explored in the previous study, [25–30] the use of benzothiophene has not been examined so far. Herein we describe an efficient and simple method for the preparation of acyl benzothiophenes (**3** and **4**) that involves the first use of benzothiophene in the aromatic acylation process mediated by the mixed anhydrides of trifluoroacetic acid (Scheme 1). The results of this study are summarized in Table 1.

**Table 1:** Synthesis of acyl substituted benzothiophenes (**3** &**4**) *via* acylation of benzothiophene (**1**) with alkyl/aryl acids

Entry	RCO_2_H (**2**) RCO =	Products (**3** & **4**)		% Yield (**3**:**4**)

1	-COCH_3_	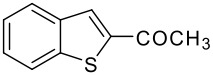		70 (1:9)
		**3a**	+	
		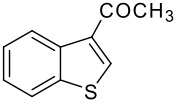		
		**4a**		
2	-COEt	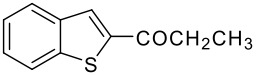		73 (1:9)
		**3b**	+	
		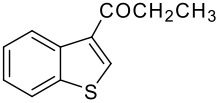		
		**4b**		
3	-CO(CH_2_)_3_CH_3_	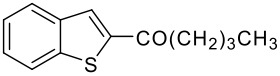		68 (1:9)
		**3c**	+	
		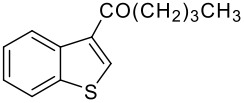		
		**4c**		
4	-CO(CH_2_)_4_CH_3_	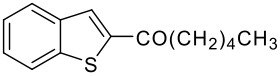		67 (1:3)
		**3d**	+	
		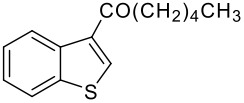		
		**4d**		
5	-CO(CH_2_)_6_CH_3_	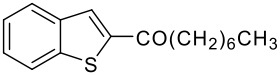		78 (2:3)
		**3e**	+	
		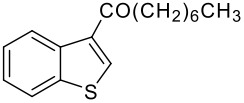		
		**4e**		
6	-COC_6_H_5_	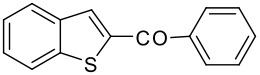		80 (1:9)
		**3f**	+	
		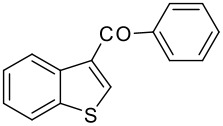		
		**4f**		
7	-COC_6_H_4_Cl-*p*	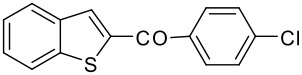		73 (2:3)
		**3g**	+	
		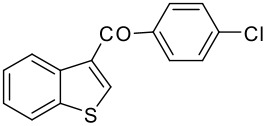		
		**4g**		
8	-COC_6_H_4_OMe-*p*	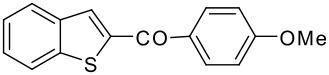		75 (2:3)
		**3h**	+	
		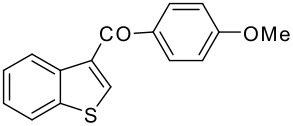		
		**4h**		
9	-COC_6_H_4_NO_2_-*p*	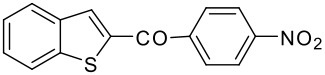		78 (2:3)
		**3i**	+	
		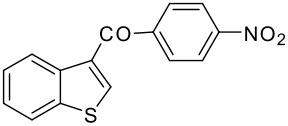		
		**4i**		

## Results and Discussion

Initially, we conducted the acylation reaction of benzothiophene with acetic acid in the presence of 85% phosphoric acid and excess trifluoroacetic anhydride (TFAA) at room temperature (25–30°C) for 30 min where no significant product formation was observed. Then the reaction was continued for a longer time and the progress of the reaction was monitored by TLC over time. This exercise resulted in isolation of acylated products and the best results were observed when the reaction was carried out for 4 h. However, a mixture of 2- and 3-acylated benzothiophene was always isolated in all these cases. An attempt to prepare a single regioisomer by varying all the reaction parameters failed perhaps due to the high reactivity of the benzothiophene ring under the conditions studied. A similar observation was noted during acetylation[16,23] of benzothiophene using i) acetic anhydride in the presence of BF_3_*Et_2_O or ii) acetyl chloride in the presence of AlCl_3_. In a typical procedure, to a mixture of commercially available acid **1** (2 g, 15 mmol) in TFAA (8.5 mL, 60 mmol) was added acetic acid (0.9 g, 15 mmol) dropwise followed by 85% H_3_PO_4_ (1.46 g, 15 mmol) with vigorous stirring at 0°C. The mixture was then warmed to 25–30°C, stirred for 4 h and poured into ice-cold water (25 mL) with vigorous stirring. The solid separated was filtered, washed with petroleum ether (2 × 5 mL) and dried to give the mixture of product **3a** and **4a** in 70 % overall yield. Based on HPLC data, [see Supporting Information File 1 for full experimental data] the isolated solid was found to be a 1:9 mixture of **3a** and **4a**. After separating these compounds using column chromatography, the structures of **3a** and **4a** was confirmed by spectral data [see Supporting Information File 1 for full spectral data]. Having prepared **3a** and **4a** successfully we then tested the reactivity of other carboxylic acids under these reaction conditions and subsequently, a variety of acylated benzothiophenes were prepared [see Supporting Information File 1 for full spectral data] (Table 1). All the reaction was carried out using **1** (1 equiv), acid **2** (1 equiv), 85% H_3_PO_4_ (1 equiv) and TFAA (4 equiv) at 25–30°C for 4–5 h and the % yields shown in Table 1 represents isolated overall yields of products. All the products were identified by ^1^H NMR, IR and mass spectra. As shown in Table 1, both aliphatic and aromatic acids participated in the acylation reaction of benzothiophene (entries 1–5 and 6–9). Aryl acids bearing an electron donating group such as methoxy (entry 8, Table 1) or electron withdrawing group such as nitro (entry 9, Table 1) afforded the desired products in good yields. While a mixture of 2- and 3-acylated benzothiophene was isolated in all the cases (entries 1–9, Table 1), the 3-isomer was isolated as the major product irrespective of the nature of the carboxylic acid used. The ratio of regioisomers present in the crude product isolated from the reaction mixture was determined by analyzing the corresponding ^1^H NMR data. The C-3 proton of compound **3** appeared in the region of 7.90–7.95 δ (when R = alkyl) and 7.97–8.0 δ (when R = aryl) whereas C-4 proton of compound **4** appeared in 8.27–8.29 δ (when R = alkyl) and 8.40–8.60 δ (when R = aryl).

We have developed a very simple and single-step process for the synthesis of acyl benzothiophenes. All the starting materials are commercially available and can be used directly. The process does not require the use of additional organic solvent but involves use of excess TFAA. While the excess of TFAA and TFA (trifluoroacetic acid produced during the reaction) was removed by treating the reaction mixture with water, their removal by distillation is more appropriate for large scale preparations.[31] This also allows the recovery of spent TFAA, as TFA can be converted back to TFAA via dehydration[32] thereby eliminating the acid waste. It is known that TFAA is produced commercially via dehydration of TFA by using SO_3_ as a dehydrating agent. However, P_2_O_5_ is more convenient in small scale preparation of TFAA.

**Scheme 2:**
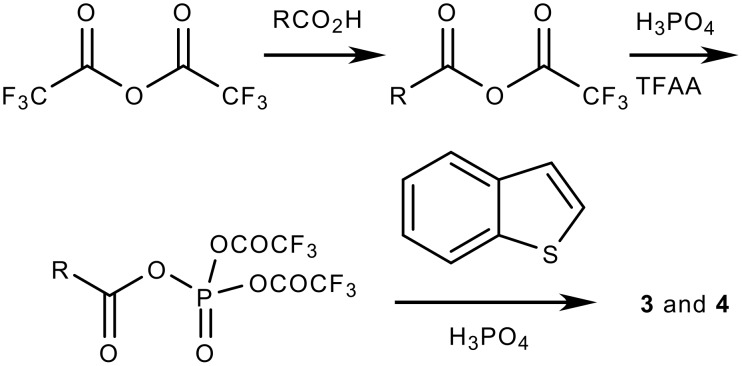
Probable mechanism for the acylation of benzothiophene

A probable mechanism for the acylation of benzothiophene is shown in Scheme 2.[25] Accordingly, phosphoric acid plays a role of covalent catalyst which leads to the generation of acyl bis(trifluoroacetyl)phosphate from the acylation precursor acyl trifluoroacetate generated *in situ*.[33] The acyl bis(trifluoroacetyl)phosphate then acetylates the benzothiophene ring in the presence of phosphoric acid to afford the products **3** and **4**. Phosphoric acid as a source of proton helps in activation. The isolation of compound **4** as the major product can be explained by the higher reactivity of C-3 over C-2 of the benzothiophene ring.

## Conclusion

In conclusion, we have described a general synthesis of a variety of acylated benzothiophenes via acylation of benzothiophene rings with in situ generated arylacetyl trifluoroacetates. The reaction proceeds at room temperature and does not involve the use of any expensive reagents or catalysts. Other advantages of the present protocol include i) ready availability of the starting materials and mild reaction conditions, ii) environmentally safe as the protocol is free from the use of inorganic Lewis acids as well as chlorinated hydrocarbons as solvent, iii) simple operational procedure. However, formation of a mixture of regioisomers is the major drawback of this protocol. Nevertheless, the present process is certainly superior to the classical Friedel-Crafts acylation technique and other multi step synthesis. Further studies on establishing the regeioselectivity and application of this methodology in organic synthesis are under investigation.

## Supporting Information

File 1 Transition-metal/lewis acid free synthesis supporting info. HPLC conditions and spectral data for compounds **3a** and **4a**, spectral data for other selected compounds (**3e**, **3f** and **4f**).
