# Supplementary material for: Transition-metal/Lewis acid free synthesis of acyl benzothiophenes via C-C bond forming reaction
Source: Beilstein J Org Chem. 2007 Oct 25;3:35. doi: 10.1186/1860-5397-3-35 (PMC2200667; doi:10.1186/1860-5397-3-35)
Supplement: File 1 — Transition-metal/lewis acid free synthesis supporting info. HPLC conditions and spectral data for compounds 3a and 4a, spectral data for other selected compounds (3e, 3f and 4f). [file Beilstein_J_Org_Chem-03-35-s001.doc]

**Transition-metal / lewis acid free synthesis supporting info.**

HPLC conditions and spectral data for compounds **3a** and **4a**,

(a) HPLC conditions (column, mobile phase (range used), flow rate, detection wavelength, retention times): Luna C8 (150 x 4.6) mm, mobile phase A: 0.05% TFA in water, mobile phase B: 0.05% TFA in CH_3_CN, gradient (T/% B) 0/10, 25/80, 25.1/10, 30/10, flow rate 1.5 mL/min, UV 230 nm, retention time 16.78 min for **3a**, retention time 17.48 min for **4a**.

(b) Spectral data for compound **3a**: ^1^H NMR (200 MHz, CDCl_3_) δ 2.67 (s, 3H), 7.38-7.45 (m, 2H), 7.84-7.87 (m, 2H), 7.90 (s, 1H); ^13^C NMR (50 MHz, CDCl_3_) δ 191.9 (C=O), 143.6, 142.2, 138.8, 129.5, 127.1, 125.4, 124.6, 122.6, 26.7 (CH_3_); IR (cm^-1^, KBr) 3058, 2923, 1662, 1510; m/z (ES Mass) 177.0 (M+1, 50%), 161 (100%); compound **4a**: ^1^H NMR (200 MHz, CDCl_3_) δ 2.65 (s, 3H), 7.43-7.46 (m, 2H), 7.84-7.88 (m, 1H), 8.28 (s, 1H), 8.74-8.79 (m, 1H); ^13^C NMR (50 MHz, CDCl_3_) δ 192.7 (C=O), 139.6, 138.8, 137.3, 136.2, 135.1, 125.7, 125.1, 121.9, 26.4 (CH_3_); IR (cm^-1^, KBr) 2954, 2924, 1665; m/z (ES Mass) 177.0 (M+1, 100%).

Spectral data for other selected compounds, compound **3c**: ^1^H NMR (200 MHz, CDCl_3_) δ 0.97 (t, *J* = 7.3 Hz, 3H), 1.39-1.60 (m, 2H), 1.75-1.85 (m, 2H), 3.01 (t, *J* = 7.3 Hz, 2H), 7.39-7.49 (m, 2H), 7.85-7.91 (m, 2H), 7.96 (s, 1H); ^13^C NMR (50 MHz, CCl_3_) δ 194.9 (C=O), 143.9, 142.4, 139.1, 128.7, 127.2, 125.8, 124.9, 122.9, 38.9, 26.8, 22.4, 13.8 (CH_3_); IR (cm^-1^, KBr) 2933, 2951, 1659, 1590; m/z (ES Mass) 236.0 (M^+^, 100%); compound **3f**: ^1^H NMR (200 MHz, CDCl_3_) δ 7.34-7.55 (m, 5H), 7.80-7.88 (m, 4H), 7.97 (s, 1H); IR (cm^-1^, KBr) 2920, 1644, 1598; m/z (ES Mass) 239.0 (M^+^, 100%); compound **4f**: ^1^H NMR (200 MHz, CDCl_3_) δ 7.32-7.55 (m, 5H), 7.79-7.89 (m, 4H), 8.55-8.59 (m, 1H); IR (cm^-1^, KBr) 3016, 2928, 1645, 1598; m/z (ES Mass) 239.0 (M^+^, 100%).
